# Supplementary material for: Circulating Tumour Cell Associated MicroRNA Profiles Change during Chemoradiation and Are Predictive of Response in Locally Advanced Rectal Cancer
Source: Cancers (Basel). 2023 Aug 20;15(16):4184. doi: 10.3390/cancers15164184 (PMC10452825; doi:10.3390/cancers15164184)
Supplement: Supplementary file 1 [file cancers-15-04184-s001.zip › cancers-2491644-supplementary.pdf]

<u>Supplementary material</u>

Circulating tumour cell and lymphocyte associated microRNA profiles are predictive and prognostic during chemoradiation in locally advanced rectal cancer

Supplementary Figure S1: Quantile normalisation method on raw and quantile normalisation in filtered data.

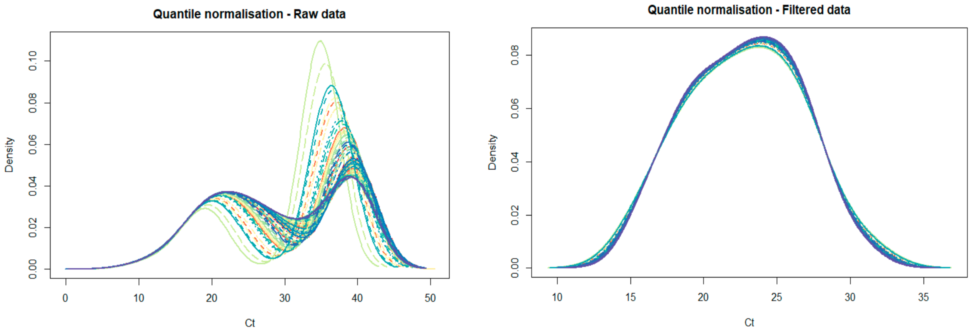

Supplementary Figure S2: Heatmap of quantile normalized microRNA data. The first two letters in the sample name identify the patient, the number that follows identifies the condition (1- Baseline; 2 – During treatment; 3 – Post-treatment). The following codes (TEV, VCE and YTK) identify the batch of the samples, ie. the time of sample collection and analysis. This shows that the batch effect is still present and needs to be addressed during the differential expression analysis.

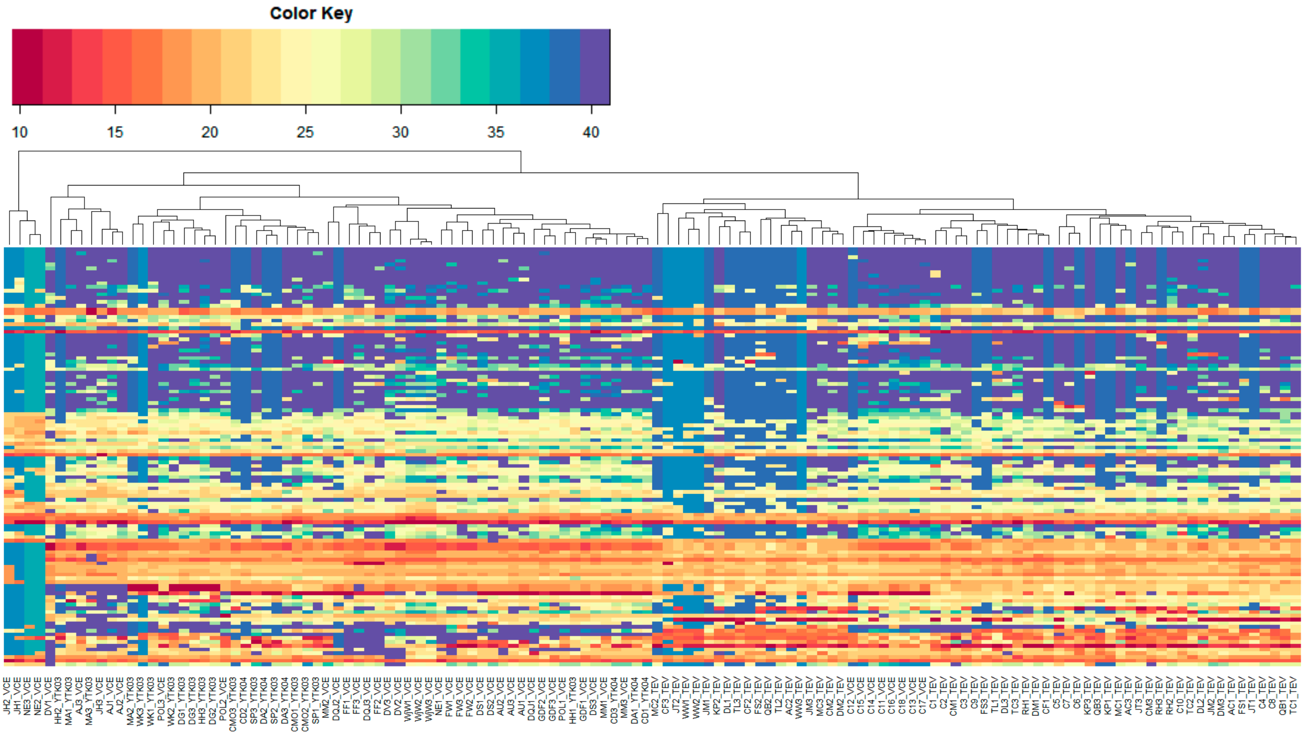

Supplementary Table S1: Circulating tumour cell count of the 52 patients at baseline, week 3 and post treatment. These time points have 1, 4, 11 samples missing respectively.

CTC (per 9ml) baseline	CTC (per 9ml) week 3	CTC (per 9ml) post treatment
12	1	8
2	1	0
11	2	1
5	1	1
1	1	
45	1	
1	4	2
4	0	0
15	1	15
0	16	2
0		
0	0	9
6	0	0
0	3	3
0	1	1
2	1	0
5	0	0
16	1	2
0	0	0
7	54	2
5	32	
19	0	40
1	0	19
1	0	1
9	0	3
0	3	6
14	9	2
5	1	8
32	4	
31	0	
0	5	
6	1	18

23		
0	10	52
15		1
3	9	1
10	0	
	11	0
1		2
0	1	4
14	0	0
2	0	1
0	1	0
2	1	
1	34	23
0	9	4
0	0	2
1	0	
10	0	4
0	0	0
8	21	1
9	0	1

Supplementary Table S2: Identification of 106 miRNA candidates (*R = Literature review associated with LARC treatment, C = Literature review associated with colorectal cancer, I = data on radiation sensitivity, and P = identified in preliminary study).

**miRNA (in numerical order)**	**Identifi-cation method***	**Summary of miRNA in previous publication(s) with reference or derived from miRBase data**
Let-7a	R	Downregulated in rectal cancer tissue compared to normal mucosa [1]
let-7c	R	Upregulation correlated with TRG 1-2 (Mandard classification) [2]
Let-7f	R	Downregulated in rectal cancer tissue compared to normal mucosa [1]
miR-100	R	Downregulated in rectal cancer tissue compared to normal mucosa [1]

miR-101	R	Downregulated in rectal cancer tissue compared to normal mucosa [1]
miR-106a	R	Upregulated in rectal cancer tissue [3]
miR-107	C, I	Upregulated in colorectal cancer tissue and cell lines when compared to normal; exerts a positive role in the survival of cancer cells by directly targeting Par4 [4]
miR-10a	R	Upregulated in rectal cancer tissue [3]
miR-10b#	R	Downregulated in rectal cancer tissue compared to normal mucosa [1]
miR-1183	R	Upregulated in TRG1 patients compared to TRG>1 (Mandard classification) [5]
miR-125a-3p	R	Upregulated in TRG1 patients compared to TRG>1 (Mandard classification) [5]<br><br>Downregulation correlated with TRG 4 (Mandard classification) [2]
miR-125b1	R	Upregulation correlated with TRG 1-2 (Mandard classification) [2]
miR-126	P	Expressed only in endothelial cells, throughout capillaries as well as larger blood vessels, and acts upon various transcripts to control angiogenesis
miR-127-3p	R	Downregulated in non-responders (Mandard classification) [6]
miR-1274a	R	Upregulated in rectal cancer tissues [3]
miR-130a and miR-130b	P	Expressed in the hematopoietic stem/progenitor cell compartment but not in mature blood cells
miR-133a	P	Represses the expression of non-muscle genes
miR-135b	C	Upregulated in colorectal cancer tissues and plasma [7]
miR-137	P	Implicated as a tumour suppressor in colorectal cancer, squamous cell carcinoma and melanoma via cell cycle control
miR-143	R, C	Upregulated with treatment in rectal cancer [8]<br>Expression of *miR-143* in colorectal cancer tissues was significantly lower than in normal tissues [9]
miR-145	R, C	Upregulated with treatment in rectal cancer [8]; correlated with tumour regression (Schneider et al).

		Expression in colorectal cancer tissues was significantly lower than in normal tissues [9]
miR-146a	C	Involved in the regulation of inflammation and other processes that function in the innate immune system
miR-148a	R	Downregulated in rectal cancer tissue compared to normal mucosa [1]
miR-148b	C, I	Downregulated in colorectal cancer tissues and three cell lines compared with normal tissues, may act as a tumor suppressor [10]
miR-152	P	Related to miR-148
miR-15a, miR-15a#, miR-15b and miR-15b#	P	miR-15a/16-1 microRNA cluster functions as a tumour suppressor, with the oncogene BCL2 as its target
miR-16	R	Predicted complete versus incomplete response (Dworak classification) [11]
miR-16-1#	P	miR-16, miR-15, mir-195 and miR-457 are related microRNA precursor sequences from the mir-15 gene family
miR-17-3p	C	Upregulated in colorectal cancer tissues and plasma [7]
miR-17-5p	C	Upregulated in coloectal cancer tissues compared to normal tissues [9]
miR-181c	P	Central role in malignant transformation, as tumor suppressor genes
miR-183	C	Upregulated in colorectal cancer tissues compared to normal tissues [9]
miR-188-3p	C, I	Prognostic factor in colorectal cancer patients, may be due to effect on MLLT4 expression and migration of cancer cells [12]
miR18a	C	Upregulated in colorectal cancer tissues compared to normal tissues [9]
miR190b	R	Upregulated in non-responders (Mandard classification) [13]
miR-191	P	Dysregulated in a large number of different types of human tumours, including those of colorectal, breast and prostate cancers
miR-192*	R	Upregulated in responders (Mandard classification) [14]

miR-193b	P	
miR-194	R	Downregulated in rectal cancer tissue compared to normal mucosa [1]
miR-195	R	Downregulated in rectal cancer tissue compared to normal mucosa [1]
miR-19a	R	Upregulated in rectal cancer tissues [3]
miR-19b	I	Expression in cell lines increased with ionising radiation [15]
miR-200a	R	Decreased in non-responders (Mandard classification) [6]<br><br>Upregulated in responders (Mandard classification) [14]
miR-200b*	R	Upregulated in responders (Mandard classification) [14]
miR-20a	C	Upregulated in cancer tissues compared to normal tissues [9]
miR-20a#	P	Implicated in a wide variety of malignancies and are sometimes referred to as oncomirs
miR-20b	I	Expression in cell lines increased with ionising radiation [15]
miR-21	R	Upregulation correlated with TRG 1-2 (Mandard classification) [2]<br><br>Downregulation correlated with TRG 4, downregulated with treatment[8]
miR-210	R	Upregulated in responders (Mandard classification) [14]
miR-213	P	Downregulated in many tumors and thus appear to function as tumour suppressor genes
miR-215	R	Upregulated in non-responders (Mandard classification) [13]<br><br>*Conflicting findings* - upregulated in responders (Mandard classification) [14]
miR-218	P	Silenced by DNA methylation, tumour suppressing qualities
miR-22#	P	
miR-221	C	Overexpression a significant prognostic factor for poor overall survival in colorectal cancer patients and correlated with p53 expression [16]

miR-221#	P	Targets CD117, which then prevents cell migration and proliferation in endothelial cells, thus acting as an anti angiogenic miRNA
miR-222	C	Upregulated in colorectal cancer tissues and plasma [7]
mir-223	R	Upregulation associated with response [17]
miR-223#	R	Downregulated in rectal cancer tissue compared to normal mucosa [1]
miR-24	P	Suppresses expression of two crucial cell cycle control genes, E2F2 and Myc in hematopoietic differentiation
miR-25	P	
miR-26a	R	Downregulated in rectal cancer tissue compared to normal mucosa [1]
miR-26b	R	Upregulated in responders (Mandard classification) [14]
miR-27a	P	
miR-296	P	Regulates angiogenesis, the process of growth and creation of new blood vessels. It achieves this by targeting HGS mRNA, reducing its expression in endothelial cells which then results in greater number of VEGF receptors. miR-296 has predicted target sites in the transcription factor NANOG and may also contribute to carcinogenesis by dysregulating p53.
miR-29b-2	R	Upregulated in non-responders (Mandard classification) [13]
miR-29c	R	Upregulated in responders (Mandard classification) [14]
miR-30d#	P	Possibility that familial breast cancers may be caused by variation in this miRNA
miR-30e	R	Downregulated in non-responders (Mandard classification) [6]
miR-31	C	Upregulated in colorectal cancer tissues compared to normal tissues [9]
miR-342-3p	R	Downregulated in rectal cancer tissue compared to normal mucosa [1]
miR-34a	I	Expression in cell lines increased with ionising radiation [15]
miR-34a#	P	Part of the p53 tumor suppressor network, dysregulation is involved in the

and miR-34b		development of some cancers
miR-365	P	
miR-375	R	Downregulated in rectal cancer tissue compared to normal mucosa [1]
miR-378	R	Downregulated in non-responders (Mandard classification) [6]
miR-409-3p	R, C	Upregulated in non-responders (Mandard classification) [6]<br><br>miR-409-3p yielded high diagnostic accuracy in discriminating colorectal cancer from normal [18]
miR-429	R	Downregulated in rectal cancer tissue compared to normal mucosa [1]
miR-451	P	Regulates the drug-transporter protein P-glycoprotein, potentially promoting resistance to the chemotherapy drugs
miR-483-5p	R	Upregulated in TRG1 patients compared to TRG>1 (Mandard classification) [5]
miR-492	R	Upregulated in rectal cancer tissues [3]
miR-520f	P	
miR-542-3p	R	Downregulated in rectal cancer tissue compared to normal mucosa [1]
miR-590-5p	R	Predicted complete versus incomplete response (Dworak classification) [11]
miR-622	R	Upregulated in TRG1 patients compared to TRG>1 (Mandard classification) [5]
miR-628-5p	P	
miR-629	P	
miR-630	R	Upregulated in TRG1 patients compared to TRG>1 (Mandard classification) [5]<br><br>*Conflicting findings* - Downregulated in responders (Mandard classification) [14]
miR-652	R	Downregulated in rectal cancer tissue compared to normal mucosa [1]
miR-671-3p	P	

miR-7	R, C	Upregulated in responders (Mandard classification) [14]<br><br>miR-7 yielded high diagnostic accuracy in discriminating colorectal cancer from normal [18]
miR720	R	Downregulated in TRG1 patients compared to TRG>1 (Mandard classification) [5]
miR-765	R	Upregulated in TRG1 patients compared to TRG>1 (Mandard classification) [5]
miR-9	P	A number of specific targets of miR-9 have been proposed, including the transcription factor REST and its partner CoREST
miR-92a	R	Upregulated in rectal cancer tissues [3]
miR-92a-1#	R	Upregulated in rectal cancer tissues [3]
miR-93	C	miR-93 yielded high diagnostic accuracy in discriminating colorectal cancer from normal [18]
miR-95	C	Upregulated in colorectal cancer tissues and plasma [7]
miR-98	P	miR-98 microRNA precursor is a let-7 family member
miR-let-7b	R	Downregulated in rectal cancer tissues [3]
miR-let-7d	I	Expression in cell lines increased with ionising radiation [15]
miR-let-7e	R	Upregulated in rectal cancer tissues [3]
miR-let-7g#	R, I	Significantly correlate with sensitivity to chemoradiation in cell lines, High expression associated with a good prognosis in rectal cancer patients [19]

References

1. Gaedcke, J., M. Grade, J. Camps, R. Sokilde, B. Kaczkowski, A.J. Schetter, M.J. Difilippantonio, C.C. Harris, B.M. Ghadimi, S. Moller, et al., The rectal cancer microRNAome--microRNA expression in rectal cancer and matched normal mucosa*. Clin Cancer Res,* **2012**. 18(18): p. 4919-30.
2. Bandres, E., F. Arias, D. Guerrero, I. Lopez, M. Gonzalez-Huarriz, M.L.G. Dorronsoro, M. Montes, F. Monzon, N. Torrea, P. Armendariz, et al., Association between a specific miRNA

signature and pathological response to neoadjuvant chemoradiotherapy (CRT) in locally advanced rectal cancer (LARC) patients, in ASCO Annual Meeting. 2012.

3. Li, X., G. Zhang, F. Luo, J. Ruan, D. Huang, D. Feng, D. Xiao, Z. Zeng, X. Chen, and W. Wu, Identification of aberrantly expressed miRNAs in rectal cancer. *Oncol Rep,* **2012**. 28(1): p. 77-84.
4. Liu, F., S. Liu, F. Ai, D. Zhang, Z. Xiao, X. Nie, and Y. Fu, miR-107 Promotes Proliferation and Inhibits Apoptosis of Colon Cancer Cells by Targeting Prostate Apoptosis Response-4 (Par4). *Oncol Res,* **2017**. 25(6): p. 967-974.
5. Della Vittoria Scarpati, G., F. Falcetta, C. Carlomagno, P. Ubezio, S. Marchini, A. De Stefano, V.K. Singh, M. D'Incalci, S. De Placido, and S. Pepe, A specific miRNA signature correlates with complete pathological response to neoadjuvant chemoradiotherapy in locally advanced rectal cancer. *Int J Radiat Oncol Biol Phys,* **2012**. 83(4): p. 1113-9.
6. D'Angelo, E., M. Fassan, I. Maretto, S. Pucciarelli, C. Zanon, M. Digito, M. Rugge, D. Nitti, and M. Agostini, Serum miR-125b is a non-invasive predictive biomarker of the pre-operative chemoradiotherapy responsiveness in patients with rectal adenocarcinoma. *Oncotarget,* **2016**.
7. Ng, E.K., W.W. Chong, H. Jin, E.K. Lam, V.Y. Shin, J. Yu, T.C. Poon, S.S. Ng, and J.J. Sung, Differential expression of microRNAs in plasma of patients with colorectal cancer: a potential marker for colorectal cancer screening. *Gut,* **2009**. 58(10): p. 1375-81.
8. Drebber, U., M. Lay, I. Wedemeyer, D. Vallbohmer, E. Bollschweiler, J. Brabender, S.P. Monig, A.H. Holscher, H.P. Dienes, and M. Odenthal, Altered levels of the onco-microRNA 21 and the tumor-supressor microRNAs 143 and 145 in advanced rectal cancer indicate successful neoadjuvant chemoradiotherapy. *Int J Oncol,* **2011**. 39(2): p. 409-15.
9. Motoyama, K., H. Inoue, Y. Takatsuno, F. Tanaka, K. Mimori, H. Uetake, K. Sugihara, and M. Mori, Over- and under-expressed microRNAs in human colorectal cancer. *Int J Oncol,* **2009**. 34(4): p. 1069-75.
10. Song, Y., Y. Xu, Z. Wang, Y. Chen, Z. Yue, P. Gao, C. Xing, and H. Xu, MicroRNA-148b suppresses cell growth by targeting cholecystokinin-2 receptor in colorectal cancer. *Int J Cancer,* **2012**. 131(5): p. 1042-51.
11. Kheirelseid, E.A., N. Miller, K.H. Chang, C. Curran, E. Hennessey, M. Sheehan, J. Newell, C. Lemetre, G. Balls, and M.J. Kerin, miRNA expressions in rectal cancer as predictors of response to neoadjuvant chemoradiation therapy. *Int J Colorectal Dis,* **2013**. 28(2): p. 247-60.
12. Pichler, M., V. Stiegelbauer, P. Vychytilova-Faltejskova, C. Ivan, H. Ling, E. Winter, X. Zhang, M. Goblirsch, A. Wulf-Goldenberg, M. Ohtsuka, et al., Genome-Wide miRNA Analysis Identifies miR-188-3p as a Novel Prognostic Marker and Molecular Factor Involved in Colorectal Carcinogenesis. *Clin Cancer Res,* **2017**. 23(5): p. 1323-1333.
13. Svoboda, M., J. Sana, P. Fabian, I. Kocakova, J. Gombosova, J. Nekvindova, L. Radova, R. Vyzula, and O. Slaby, MicroRNA expression profile associated with response to neoadjuvant chemoradiotherapy in locally advanced rectal cancer patients. *Radiat Oncol,* **2012**. 7: p. 195.

14. Millino, C., I. Maretto, B. Pacchioni, M. Digito, A. De Paoli, V. Canzonieri, E. D'Angelo, M. Agostini, F. Rizzolio, A. Giordano, et al., Gene and microRNA Expression Are Predictive of Tumor Response in Rectal Adenocarcinoma Patients Treated with Preoperative Chemoradiotherapy. *J Cell Physiol,* **2016**.
15. Metheetrairut, C. and F.J. Slack, MicroRNAs in the ionizing radiation response and in radiotherapy. *Curr Opin Genet Dev,* **2013**. 23(1): p. 12-9.
16. Pu, X.X., G.L. Huang, H.Q. Guo, C.C. Guo, H. Li, S. Ye, S. Ling, L. Jiang, Y. Tian, and T.Y. Lin, Circulating miR-221 directly amplified from plasma is a potential diagnostic and prognostic marker of colorectal cancer and is correlated with p53 expression. *J Gastroenterol Hepatol,* **2010**. 25(10): p. 1674-80.
17. Hotchi, M., M. Shimada, N. Kurita, T. Iwata, H. Sato, S. Morimoto, K. Yoshikawa, J. Higashijima, and T. Miyatani, microRNA expression is able to predict response to chemoradiotherapy in rectal cancer. *Mol Clin Oncol,* **2013**. 1(1): p. 137-142.
18. Wang, S., J. Xiang, Z. Li, S. Lu, J. Hu, X. Gao, L. Yu, L. Wang, J. Wang, Y. Wu, et al., A plasma microRNA panel for early detection of colorectal cancer. *Int J Cancer,* **2013**.
19. Salendo, J., M. Spitzner, F. Kramer, X. Zhang, P. Jo, H.A. Wolff, J. Kitz, S. Kaulfuss, T. Beissbarth, M. Dobbelstein, et al., Identification of a microRNA expression signature for chemoradiosensitivity of colorectal cancer cells, involving miRNAs-320a, -224, -132 and let7g. *Radiother Oncol,* **2013**. 108(3): p. 451-7.
